# Physicochemical and Thermo–Mechanical Characterization of Sheep Wool/Phenolic Novolac Panels for Sustainable Thermal Insulation

**DOI:** 10.3390/ma19122488

**Published:** 2026-06-10

**Authors:** Jakub Barwinek, Piotr Szatkowski, Julita Szczecina, Wiktoria Borowicz, Andrzej Czulak, Edyta Molik

**Affiliations:** 1 Department of Glass Technology and Amorphous Coatings, Faculty of Materials Science and Ceramics, AGH University of Krakow, Al. Mickiewicza 30, 30-059 Krakow, Poland; barwinek@agh.edu.pl (J.B.); wiktoriaborowicz@icloud.com (W.B.); 2 Department of Animal Biotechnology, Faculty of Animal Science, University of Agriculture in Krakow, Al. Mickiewicza 24/28, 31-059 Krakow, Poland; julita.szczecina@student.urk.edu.pl; 3 Łukasiewicz Research Network–Institute of Ceramics and Building Materials, 31-983 Krakow, Poland; andrzej.czulak@icimb.lukasiewicz.gov.pl

**Keywords:** sheep wool, phenolic novolac resin, bio-based insulation panels, thermal conductivity, mechanical properties, DSC, TGA, fire performance, circular economy, sustainable building envelopes

## Abstract

This study reports the physicochemical characterization and structure–property relationships of rigid sheep wool/phenolic novolac panels developed as bio-based thermal insulation for building envelopes. Mixed Polish sheep wool was washed, mechanically opened, and formed into nonwoven mats, then impregnated with either neat or flame-retardant novolac resin to obtain lightweight boards with a fiber content of about 50 wt%. Elemental analysis, ICP-OES, FTIR spectroscopy, and laser and electron microscopy were used to evaluate the fiber composition, keratin structure, morphology, and fiber–matrix interfaces. Mechanical performance under three-point bending and shear, differential scanning calorimetry, thermogravimetric analysis, and transient hot-probe thermal-conductivity measurements were applied to link microstructure with functional behavior. Novolac impregnation transformed the compliant wool mat into self-supporting panels, increasing the flexural modulus to the 0.8–1.4 GPa range and flexural strength to approximately 48–52 MPa, while the shear modulus and work to failure rose by more than an order of magnitude relative to the loose wool reference. Thermal conductivity remained in a typical range for natural-fiber insulations (λ = 0.061 W·m^−1^·K^−1^ for the wool mat and 0.071–0.074 W·m^−1^·K^−1^ for the composites), although higher than that of expanded polystyrene. DSC and TGA confirmed that wool fibers remain thermally stable up to about 200–220 °C, that the novolac resin cures around 140 °C, with typical phenolic reaction enthalpies, and that both formulations generate high char residues of roughly 60–80 wt% at 600 °C under nitrogen, evidencing a strong charring propensity rather than directly quantifying fire resistance. Overall, the results position sheep wool/novolac panels between conventional bio-based insulation and structural composites and highlight their potential as sustainable, circular insulation materials for energy-efficient building envelopes.

## 1. Introduction

The building sector is currently responsible for more than one-third of global final energy consumption and close to 40% of total direct and indirect CO_2_ emissions, making it a central focus of climate and energy policy [1,2]. Within the European Union, this has translated into increasingly stringent requirements arising from the Energy Performance of Buildings Directive (EPBD), the Renovation Wave initiative, and the European Green Deal, which together target deep reductions in operational energy demand and greenhouse-gas emissions from buildings [3]. In parallel, the new Circular Economy Action Plan and the ongoing revision of the Construction Products Regulation emphasize resource efficiency, waste reduction, and recyclability of construction materials over their entire life cycle [4]. Against this backdrop, there is a growing demand for thermal insulation solutions that not only deliver low U-values, but also minimize embodied energy and carbon, facilitate end-of-life recovery, and fit into circular value chains [5,6]. Consequently, there is a growing need for insulation materials that combine low U-values with reduced embodied energy and carbon, and that can be more easily recovered and recirculated at end-of-life.

Conventional fibrous and foam-based insulations, such as mineral wool, expanded polystyrene (EPS), and extruded polystyrene (XPS), have played a key role in improving building energy efficiency, but they also exhibit important environmental and safety limitations [7]. Their production relies largely on nonrenewable raw materials and energy-intensive processes, contributing significantly to embodied CO_2_ emissions [8]. Life-cycle studies and value-chain analyses highlight that end-of-life management of EPS and stone wool is particularly challenging: these materials are often contaminated in demolition waste streams, mechanically or chemically difficult to separate, and therefore only marginally recycled in practice, with landfill or energy recovery remaining the dominant routes [9]. Moreover, polymeric foams, such as EPS and XPS, are highly combustible; under fire exposure, they undergo rapid pyrolysis, shrinkage, and melting, producing dense smoke and toxic gases that aggravate fire spread and tenability conditions, which has led several national regulations to restrict their use or require additional fire protective layers [10]. Renewable raw materials and energy-intensive processes, contributing significantly to embodied CO_2_ emissions of the life management of EPS and stone wool, are particularly challenging: these materials are often contaminated in demolition waste streams, mechanically or chemically difficult to separate, and therefore only marginally recycled in practice, with landfill or energy recovery remaining the dominant routes for protective layers.

In this context, natural-fiber-based insulation materials have emerged as promising candidates for more sustainable building envelopes. Within the EU circular-economy framework, sheep wool is increasingly recognized not only as a renewable fiber but also as a problematic by-product, since coarse, low-value fractions often lack profitable textile applications and are therefore stockpiled, landfilled, or even burned on farms. Recent analyses for Poland and other European countries show that a substantial share of the annual wool clip is still treated as waste, despite its biogenic origin and favorable physicochemical properties. As highlighted by Szczecina et al., reclassifying such low-grade wool from waste to biomass resource is fully consistent with circular-economy goals and opens up new valorization pathways, including its use in building materials and environmental applications [11].

Among them, ovine wool is particularly attractive because it is an annually renewable byproduct of livestock farming, often available as low-value or waste wool not suitable for the textile industry and can therefore contribute to waste valorization and rural circular economies [12,13]. Thermal insulation products manufactured from sheep wool fibers typically exhibit thermal conductivity values in the range of 0.035–0.045 W·m^−1^·K^−1^, comparable to conventional mineral wool and polymer foams and fully meeting standard requirements for building insulation materials [14]. Recent experimental studies on sheep wool-based mats and composites report λ values between approximately 0.033 and 0.044 W·m^−1^·K^−1^, depending on density, binder type, and product structure, confirming that well-designed wool felts can achieve competitive thermal performance [15]. Products of livestock farming, often available as low-value or waste wool not suitable for the textile industry, can therefore contribute to waste valorization and rural circular economies.

Beyond their thermal efficiency, sheep wool fibers offer additional functional and environmental benefits that are relevant to EU circularity targets [16]. Wool-based products are largely bio-based and potentially biodegradable, with low water-vapor diffusion resistance factors that support “breathable” envelope assemblies and moisture buffering of indoor spaces [17,18]. From a fire-safety perspective, wool exhibits an inherently low flammability: its keratinous structure, relatively high nitrogen and bound water contents, and high ignition temperature (around 560 °C) result in limited flame spread, char formation, and, in many configurations, a tendency to self-extinguish once the external heat source is removed [19]. These characteristics, combined with the ability of wool to sorb certain indoor air pollutants, position ovine wool felts as multifunctional thermal insulation materials that can contribute simultaneously to energy efficiency, indoor environmental quality, and circular-economy objectives [20]. Extinguish once the external heat source is removed.

However, despite the growing technical literature on wool-based insulation systems at the product scale, the links between the physicochemical characteristics of raw ovine wool fibers and the resulting macroscopic performance of thermal insulation felts remain only partially understood [21]. Properties such as fiber diameter distribution, crimp, cuticle morphology, surface chemistry, and residual grease content can be expected to influence not only the effective thermal conductivity, but also the mechanical stability, hygroscopic behavior, and fire response of wool-based insulations [22]. A systematic physicochemical characterization of ovine wool fibers, combined with an analysis of structure–property relationships at the felt level, is therefore needed to rationally design wool-based thermal insulation materials with optimized performance and reliability, and to support their wider implementation in energy-efficient, low-impact building envelopes [23].

## 2. Materials and Methods

### 2.1. Research Methods

The contents of nitrogen (N) and carbon (C) in the wool samples were determined using a vario MAX cube elemental analyzer (Elementar Analysensysteme GmbH, Langenselbold, Germany), based on catalytic high-temperature combustion of organic samples in an oxygen atmosphere, followed by quantitative detection of the evolved gases.

For the determination of other elements, approximately 0.5 g of wool was weighed into the vessels of a closed microwave digestion system. Wet digestion was carried out with a mixture of 1 mL hydrogen peroxide (H_2_O_2_) and 7 mL nitric acid (HNO_3_). The samples were mineralized in a Multiwave 5000 microwave digestion system equipped with a 41HVT56 rotor (Anton Paar GmbH, Graz, Austria) using the following program: ramp time 15 min to 190 °C, hold time 25 min at 190 °C, maximum power 1800 W, and maximum pressure 40 bar. After cooling, the digests were filtered into 25 mL volumetric flasks and diluted to volume with deionized water. Elemental concentrations in the digested solutions were determined by inductively coupled plasma optical emission spectrometry (ICP-OES), following the procedure described in PN-R-04014:1991: “Chemical and agricultural analysis of plants–Methods of mineralization of plant material for the determination of macro- and microelements”. ICP-OES was chosen because it enables simultaneous multi-element determination in digested organic matrices with high sensitivity and precision. The mechanical behavior under compression and three-point bending was evaluated for three wool-based materials: a pure sheep wool specimen, a composite of sheep wool with neat novolac resin, and a composite of sheep wool with novolac resin containing the MD3_54 flame retardant. Cylindrical specimens with a diameter of 38 mm and a height of 17 mm were prepared for each material variant, and two repetitions were carried out per material to ensure the repeatability of the results. Compressive strength tests were performed using a universal testing machine, Static Testing System type 1445 RetroLine Zwick/Roell (Ulm, Germany), equipped with suitable compression platens and dedicated control software. Cylindrical specimens with a diameter of 38 mm and a height of 17 mm were prepared for each material variant, and two repetitions were carried out per material to ensure repeatability of the results. Compressive tests were carried out in displacement control at a crosshead speed of 2 mm/min, and the specimens were compressed uniaxially up to 80% reduction in their initial height. The measurement protocol followed the general recommendations of PN-EN ISO 844 for compression testing of rigid cellular plastics, adapted to the present fibrous composite geometry.

Flexural properties were investigated using a Zwick/Roell testing machine coupled with testXpert III v1.6 software (ZwickRoell GmbH & Co. KG, Ulm, Germany) installed on a desktop computer. In the three-point bending configuration, the specimen of the appropriate length was placed on two supports and loaded at mid-span, with a normal force applied perpendicularly to its surface by means of a loading nose. The test was continued until decohesion and failure of the specimen occurred, and the recorded data were used to calculate flexural strength, flexural strain, and Young’s modulus in bending. The three-point bending tests were carried out in accordance with the PN-EN ISO 178:2019 standard for the determination of flexural properties of plastics, applying a crosshead speed of 2 mm/min for the flexural modulus determination phase and 10 mm/min for the subsequent flexural strength measurement phase.

Thermogravimetric analysis (TGA) was performed to characterize the thermal stability, moisture content, and resin curing behavior of the prepared composite samples using a TA Instruments TGA 550 Discovery thermogravimetric analyser (TA Instruments, New Castle, DE, USA). Approximately 10–15 mg of each sample was placed in an open platinum pan and analyzed under a high-purity nitrogen atmosphere (flow rate: 50 mL/min) over a temperature range of 45–600 °C, applying a constant heating rate of 10 °C/min. The mass loss percentage was quantified at key temperatures: 100 °C (corresponding to moisture evaporation) and 170 °C (associated with the resin curing/maturation stage). The onset temperature of thermal degradation was determined as the temperature at which 1% mass loss occurred, providing a reliable indicator of the composite’s initial decomposition threshold. All measurements were conducted in triplicate to ensure reproducibility, with data processed using TA Instruments TRIOS v5.8 software (TA Instruments, New Castle, DE, USA). for baseline correction and derivative thermogravimetric (DTG) analysis.

Differential scanning calorimetry (DSC) was carried out using a DSC 1 instrument (Mettler Toledo, Greifensee, Switzerland). The calorimeter was equipped with an Intra Cooler unit to enable controlled sample cooling. Instrument operation and data processing were performed using the STARe Thermal Analysis Software, version 16.20. Using this setup, conventional dynamic DSC scans were obtained for all materials, and additional isothermal experiments were conducted for selected formulations to evaluate crystallization kinetics.

It should be emphasized that DSC was not used here as a primary, quantitative method for determining water absorption. Standardized assessment of moisture uptake in polymeric and composite materials relies on gravimetric procedures, such as ISO 62 or ASTM D570, in which specimens are conditioned to constant mass before and after exposure to a controlled humid or liquid–water environment. In the present work, DSC is therefore employed only as a complementary tool to qualitatively indicate the presence and approximate temperature range of moisture-related endothermic effects, rather than to derive numerical values of water content or sorption capacity.

Following biocomposite fabrication, specimens intended for DSC were cut into small fragments (approximately 5–10 mg) and conditioned prior to analysis. Storage conditions were controlled as follows: a temperature of 23 ± 2 °C and a relative humidity of 50 ± 5%. Depending on the experimental variant, samples were either kept in sealed glass vials with silica gel desiccant or exposed to ambient air to allow moisture equilibration. Owing to the strongly hygroscopic nature of sheep wool (it can bind up to about 35% water relative to its dry mass), rigorous conditioning was necessary; otherwise, the endothermic effects associated with water evaporation in DSC would predominantly reflect variations in residual moisture content rather than intrinsic characteristics of the biocomposite.

The DSC experiments were conducted in aluminum crucibles with a nominal volume of 40 μL. Pans were crimped for heating runs and used with open lids during cooling segments. The sample mass in each crucible was between 5 and 10 mg and was determined gravimetrically using an analytical balance, with an accuracy of ±0.1 mg. Measurements were performed in a temperature range from −25 °C to 300 °C during heating and from 300 °C down to 25 °C during cooling. A constant heating and cooling rate of 10 °C/min was applied, in line with typical conditions for biocomposite analysis as specified in ISO 11357-1. All scans were carried out under an inert nitrogen atmosphere with a flow rate of 50 mL/min to minimize oxidative degradation during thermal treatment. Before DSC analysis, all specimens were oven-dried at (105 ± 2) °C to constant mass, and subsequently cooled in a desiccator to minimize moisture-related artifacts in the thermograms.

Fourier transform infrared (FTIR) spectroscopy was carried out using a Bruker Tensor 27 spectrometer (Bruker Optik GmbH, Billerica, MA, U.S.) to investigate the chemical structure of the materials. This technique was applied to mixed washed sheep wool fibers, as well as to composite samples consisting of sheep wool and novolac resin. The measurements were performed using the attenuated total reflectance (ATR) mode with a diamond crystal, which enables direct analysis of solid samples without complex preparation and provides spectra representative of the near-surface region of the material.

Spectra were recorded in the mid-infrared range from 4000 to 400 cm^−1^ with a spectral resolution of 4 cm^−1^, and 64 scans were accumulated for each measurement to improve the signal-to-noise ratio and ensure good spectral quality. Prior to each series of measurements, a background spectrum was collected under the same conditions and automatically subtracted from the sample spectra to eliminate atmospheric contributions, such as water vapor and carbon dioxide. The applied FTIR-ATR configuration allowed for the identification of characteristic functional groups present in the wool fibers and in the wool/novolac composites, and thus for the assessment of possible chemical interactions or structural modifications induced by the incorporation of the resin.

Microscopic examinations were performed using a Keyence VHX-900F digital microscope (Keyence International, Osaka, Japan) equipped with a versatile optical system enabling continuous magnification in the 20×–200× range. The analysis employed the instrument’s capability to generate three-dimensional reconstructions by stacking images captured at various focal depths, which ensured an extended depth of field and improved image sharpness. Digital microscopy served as a supplementary technique for verifying the structural integrity of the composites. The microscopic evaluation included quantification of void content, assessment of the fiber dispersion homogeneity, and inspection of fiber–matrix interfacial bonding. Representative micrographs at high magnification confirmed that the tested specimens conformed to the quality requirements necessary for reliable mechanical characterization.

High-resolution surface imaging of sheep wool fibers was carried out using a VK-X3000 3D Laser Scanning Microscope (Keyence International, Osaka, Japan). This system utilizes a 408 nm confocal laser source to generate precise topographic maps and detailed surface profiles with sub-micrometer resolution (vertical resolution: 0.1 µm). The instrument’s ability to combine optical and laser data allowed for accurate reconstruction of fiber morphology over scan heights up to 17.5 mm, enabling quantitative assessment of surface roughness (Ra, Rz), scale structure, and processing-induced defects. The 3D imaging mode provided a highly detailed visualization of fiber geometry, supporting the evaluation of interface quality and potential mechanical interactions between fibers and the polymer matrix.

The surface morphology of the wool fibers was examined using a Thermo Fisher Scientific Apreo 2 scanning electron microscope (Waltham, MA, USA), a high-resolution field-emission SEM designed for advanced materials characterization. This instrument enables the acquisition of nanometer-scale images at a range of working distances and operating conditions, which is particularly advantageous for imaging non-conductive, fiber-based materials, such as wool composites.

Scanning electron microscopy (SEM) is based on scanning a finely focused beam of high-energy electrons across the specimen surface, where it interacts with the atoms of the material and generates various signals, such as secondary and backscattered electrons, which are detected and converted into a high-resolution image of the sample topography and, to some extent, its composition. Owing to the shallow escape depth of secondary electrons, SEM is especially suitable for revealing fine surface features and defects on individual fibers, which was the main objective of the observations in this study.

The thermal conductivity coefficient (λ) was determined using the transient hot probe method with an ISOMET 2104 testing system (Applied Precision Ltd., Rača, Slovakia), equipped with a measurement probe optimized for low-conductivity materials in the range of 0.01–0.3 W/(m·K). Composite specimens were prepared as square plates measuring 70 × 70 mm, with a minimum thickness of 15 mm to ensure reliable heat flux penetration and accurate transient temperature response recording. Measurements were conducted under controlled ambient conditions to minimize convective interference and obtain reproducible thermal property data.

### 2.2. Materials and Production of Composite Samples

The ovine wool fibers used in this study were supplied by the University of Agriculture in Krakow (Poland), originating from the Experimental Station of the Department of Animal Biotechnology located in Bielany (Krakow district). The raw material consisted of mixed fleeces obtained as a by-product of routine shearing, representative of typical Polish meat–wool sheep breeds. Prior to composite manufacturing, the wool underwent a standardized washing and mechanical opening procedure to remove greasy impurities and to obtain a homogeneous, well-aerated fiber mass suitable for further processing.

**Figure 1:**
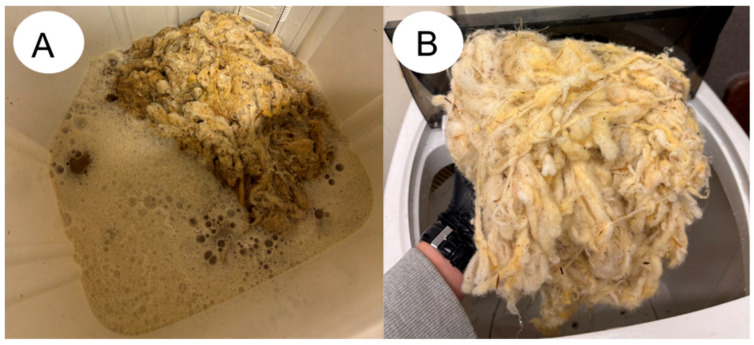
Contaminants visible during washing of mixed sheep wool (**A**), and sheep wool after washing in an automatic washing machine (**B**). (Photos by Jakub Barwinek).

The scouring process was carried out in a domestic front-loading automatic washing machine. The raw wool was placed in the drum in loosely filled mesh bags to avoid excessive felting and to facilitate water exchange. Washing was performed using a program with a water temperature of 30 °C. As a detergent, a mild, liquid grey soap of Polish origin (Biały Jeleń, Ostrzeszów, Poland) was used, added according to the manufacturer’s recommendation for delicate fabrics. The washing cycle included three consecutive stages of rinsing with fresh water to effectively remove detergent residues, dirt, and soluble contaminants, which are visible in Figure 1A. Immediately after washing, the wool was manually separated and gently fluffed to prevent matting prior to mechanical carding. A photograph of the wool after the washing process is provided in Figure 1B.

**Figure 2:**
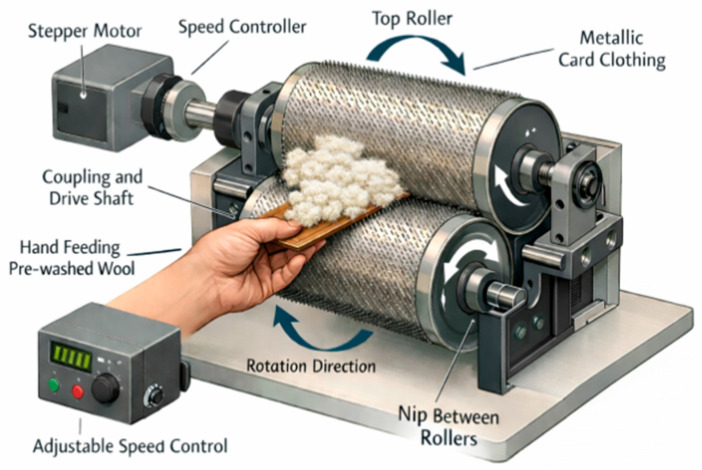
The process of carding wool on a laboratory carding machine. Own work from Jakub Barwinek, with the use of GPT-5.2 (from OpenAI).

**Figure 3:**
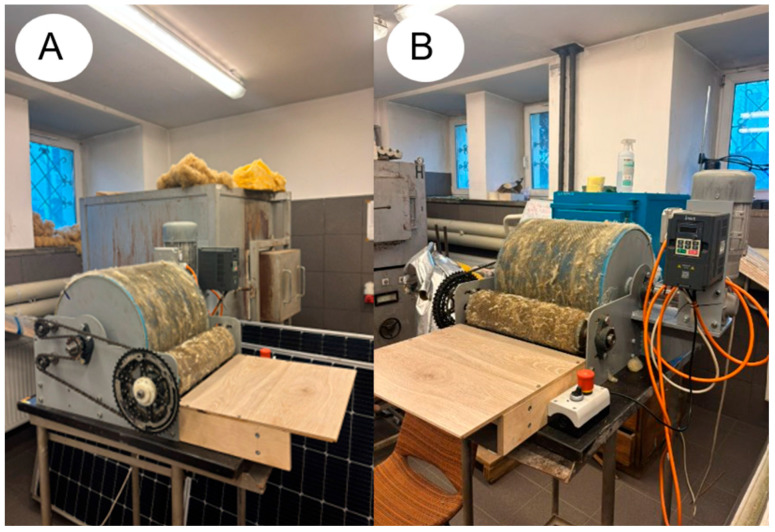
The left side of the carding device with the gear mechanism visible (**A**), and the right side of the device showing the electric motor (**B**). (Photos by Jakub Barwinek).

Mechanical opening and blending of the scoured wool were conducted using a custom-built laboratory carding device. The carder consisted of two cylindrical rollers equipped with metallic card clothing (needled surfaces), mounted on a common frame and mechanically coupled by a shaft and a clutch. The rotation of the rollers was driven by a stepper motor, allowing precise control of the peripheral speed and direction of rotation. The system was equipped with an adjustable speed controller, which made it possible to optimize the carding intensity and residence time of the fibers in the working zone. During operation, small batches of pre-washed wool were fed manually into the nip between the counter-rotating rollers until a uniformly opened and mixed fiber web was obtained. The wool carding process is shown in the graphic below (Figure 2). Furthermore, a photograph of the custom carding machine is shown in Figure 3.

The polymer matrices used for composite fabrication were novolac-type phenolic resins in powder form, supplied by LERG S.A. (Pustków, Poland). Two resin variants were applied: a pure novolac resin (without flame retardant additives) and a flame-retardant-modified novolac resin with the code MD 3_54. The flame-retardant novolac resin (MD 3_54, LERG S.A., Poland) contains a proprietary inorganic flame-retardant package optimized for phenolic foams, with a total additive content of approximately 10 wt%, according to the manufacturer’s datasheet. The detailed formulation is not disclosed, but the presence of these additives should result in an increased tendency to coke and an enhanced stability of the solid residue under anaerobic conditions. The use of these two resin systems enabled evaluation of the influence of the flame-retardant modification on the processing behavior and properties of the resulting composites. The composite panels were produced in a batch process according to the following procedure. First, the pre-carded, dry wool fibers and the novolac resin powder were weighed in a 1:1 mass ratio, corresponding to a nominal fiber content of 50 wt% in the composite formulation. Subsequently, the resin powder was pre-heated to 95 °C to decrease its viscosity and to facilitate the wetting and impregnation of the fibers. The composite panels were manufactured in a steel mold measuring 150 × 150 mm, and with a target thickness of 20 mm. After filling the mold with a mixture of fibers and resin, the assembly was pressed at 170 °C for 5 min at a pressure of 10 MPa, in accordance with the resin manufacturer’s recommendations. After curing, the panels were cooled in the mold to room temperature, and then conditioned for at least 48 h at 23 ± 2 °C and 50 ± 5% RH before mechanical and thermal testing.

**Figure 4:**
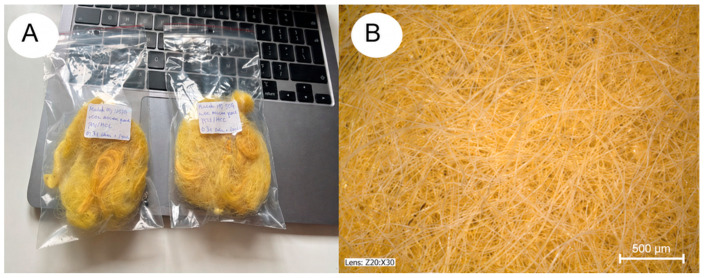
Composite samples made from sheep wool with the addition of pure novolac resin (**A**), and a photo taken with a digital microscope at 100× magnification showing wool fibers coated with resin (**B**). (Photos by Jakub Barwinek).

Crosslinking of the composite panels was carried out at a temperature of 170 °C, maintained for the time required to achieve complete curing of the novolac matrix, as specified by the resin supplier. After curing, the panels were removed from the mold and conditioned under laboratory conditions before further physicochemical and mechanical characterization. In addition, the morphology of the wool–resin composites was documented at both the macro-scale and micro-scale: photographs of the as-manufactured composite panels were taken with the naked eye to illustrate their overall appearance and homogeneity, while representative micrographs of the composite cross-sections were acquired using optical microscopy (Keyence International, Osaka, Japan) to visualize the fiber distribution, fiber–matrix adhesion, and the internal porosity of the material (Figure 4).

Figure 2 in this study was created using the GPT-5.2 (from OpenAI) for the purposes of generating graphics. The authors have reviewed and edited the output and take full responsibility for the content of this publication.

## 3. Results and Discussion

### 3.1. Chemical Composition of Wool Fibers

Table 1 and Table 2 show the results of the elemental composition of washed sheep’s wool.

The elemental composition of the washed sheep wool fibers reflects both their keratinous protein structure and the environmental conditions in which the sheep lived. The high carbon (47.8%) and nitrogen (14.82%) contents are characteristic of wool keratin, confirming that the fibers are primarily composed of proteinaceous material rich in peptide bonds and sulfur-containing amino acids. The very high sulfur level (18,315.96 mg/kg) is consistent with the presence of cysteine and cystine residues forming disulfide cross-links, which are responsible for the mechanical stability of the fiber and are considered a typical feature of wool and other keratinous tissues.

The macroelements calcium (2003.42 mg/kg), potassium (1016.68 mg/kg), magnesium (339.96 mg/kg), and phosphorus (113.07 mg/kg) occur at relatively high concentrations and can be largely attributed to the animals’ living environment, including their diet, drinking water, and contact with soil and dust in the grazing area. These biogenic elements are known to accumulate in wool as the fiber grows, and their levels are often used as indicators of the mineral supply and environmental background to which the flock is exposed. The presence of iron (65.02 mg/kg), zinc (72.75 mg/kg), and aluminum (27.23 mg/kg) at lower concentrations further supports the contribution of exogenous sources, such as soil particles, atmospheric deposition, and farm infrastructure, which may adhere to the fleece during grazing and are only partially removed during washing.

Toxic elements, namely arsenic (0.14 mg/kg) and lead (0.43 mg/kg), were detected only at very low levels, suggesting that the sheep were kept in an environment with limited heavy-metal contamination. Wool is recognized as an effective bioindicator of environmental exposure to heavy metals, because contaminants from feed, water, and air can be incorporated into the growing fiber and retained over long periods. In this context, the low arsenic and lead contents point to relatively clean grazing conditions and an absence of significant industrial or mining pollution sources in the area where the animals were raised. Overall, the elemental profile of the washed wool fibers not only confirms their proteinaceous nature but also reflects the mineral composition and pollutant load of the environment in which the sheep lived, with predominantly biogenic elements and only trace levels of hazardous metals. The remaining mass fraction of the fibers can be attributed primarily to oxygen and hydrogen bound in the organic keratin matrix, together with sulphur and the mineral macro- and microelements quantified by ICP-OES (Table 1). This is consistent with the general elemental composition of wool keratin, which comprises carbon, hydrogen, oxygen, nitrogen, and sulphur, supplemented by minor amounts of inorganic ash components originating from the animals’ diet and environment.

### 3.2. FTIR Analysis—Molecular Structure

**Figure 5:**
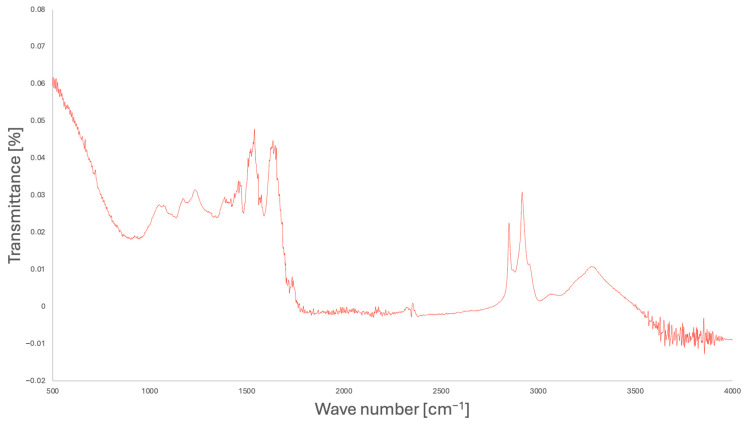
FTIR spectrum for washed mixed sheep wool.

In the FTIR spectrum of the sheep wool fibers, presented in Figure 5, a pronounced and broad absorption band is observed around 3275 cm^−1^, which is associated with overlapping N–H and O–H stretching vibrations characteristic of peptide bonds and hydrogen-bonded hydroxyl groups in the keratin structure. In the lower wavenumber region, three distinct bands appear at approximately 1635, 1508, and 1228 cm^−1^, corresponding to the amide I, amide II, and amide III regions, respectively, which are typical for protein-based materials. The amide I band arises predominantly from the stretching vibrations of the C=O groups in the peptide linkage and is highly sensitive to the secondary structure of the protein backbone. The amide II band is mainly related to N–H bending vibrations, coupled with C–N and C–H stretching modes within the polypeptide chain. The amide III band results from a more complex combination of C–N stretching and N–H bending vibrations, with additional contributions from C–C stretching and C=O deformation, collectively reflecting the conformational features of the wool keratin.

The FTIR spectrum of the uncured novolac resin exhibits a broad band at approximately 3345 cm^−1^, which can be attributed to the stretching vibrations of hydroxyl (–OH) groups associated with phenolic structures. A distinct absorption at around 2919 cm^−1^ corresponds to CH_2_ stretching vibrations, indicating the presence of methylene bridges within the polymer network.

**Figure 6:**
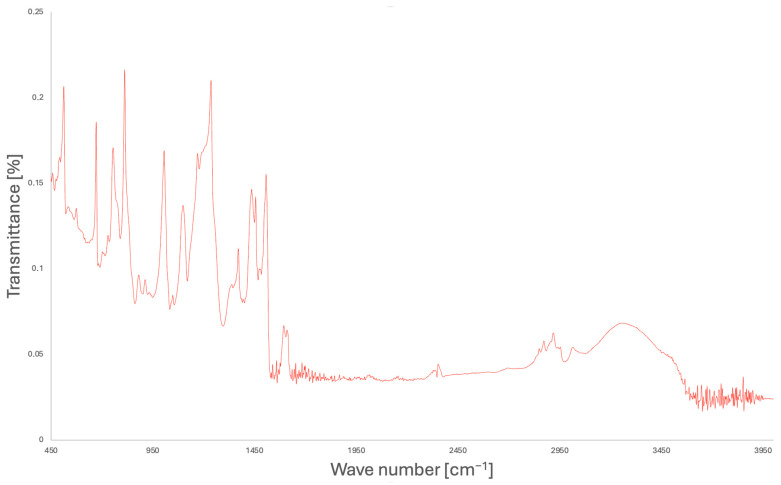
FTIR spectrum for uncured novolac resin powder.

In the aromatic region, bands observed near 1642 and 1596 cm^−1^ are assigned to C=C stretching vibrations of the benzene rings, confirming the aromatic character of the novolac backbone. An absorption peak at about 1439 cm^−1^ is related to CH_2_ bending vibrations, while a band at approximately 1361 cm^−1^ is associated with C–C double-bond deformation modes within the aromatic system. In the lower wavenumber region, bands appearing near 818 and 756 cm^−1^ are ascribed to asymmetric stretching of aromatic C–C–OH linkages and to out-of-plane C–H bending vibrations of substituted benzene rings, respectively. The overall spectral profile, presented in Figure 6, is consistent with the expected structure of phenolic novolac resins and confirms the presence of aromatic rings bridged by methylene groups and bearing hydroxyl functionalities [24].

**Figure 7:**
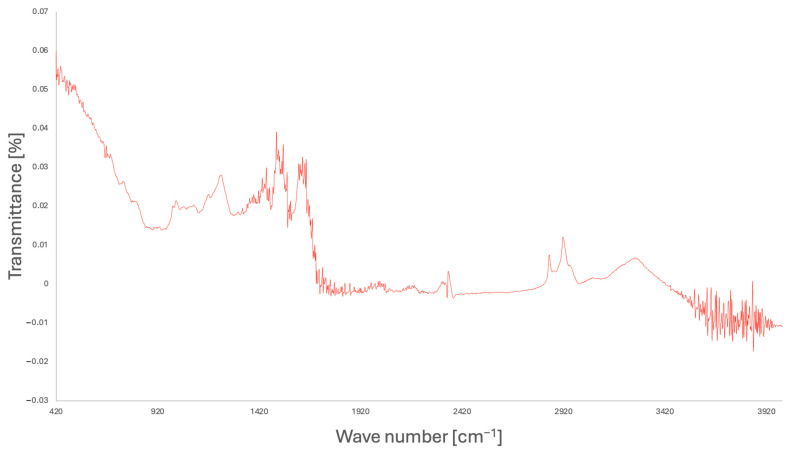
FTIR spectrum for composite material sheep wool/novolac resin powder.

In order to verify how the chemical signatures of both constituents are expressed in the final material, an FTIR-ATR spectrum was also recorded for the wool/novolac composite (Figure 7). The composite spectrum clearly combines the characteristic amide bands of the keratin fibers with the aromatic and phenolic features of the novolac matrix. In the region around 3300–3200 cm^−1^, a broad absorption is observed that reflects overlapping N–H and O–H stretching vibrations originating from peptide groups in wool and hydroxyl groups in the phenolic resin. The amide I, II, and III bands of wool remain visible near 1630, 1530, and 1230 cm^−1^, respectively, although they are partially broadened and slightly shifted compared with the spectrum of the neat fibers, which can be attributed to changes in hydrogen-bonding conditions and local chain conformation upon impregnation with the polymer.

At the same time, the composite spectrum exhibits pronounced bands associated with the novolac phase, including aromatic C=C stretching vibrations in the 1600–1500 cm^−1^ region and out-of-plane C–H bending modes of substituted benzene rings below 900 cm^−1^. The coexistence of these keratin-related and phenolic bands confirms that both components retain their fundamental chemical structure in the composite. No additional peaks characteristic of new covalent bonds between wool and resin are detected, suggesting that the fiber–matrix interaction is dominated by physical adhesion, hydrogen bonding, and mechanical interlocking rather than extensive chemical grafting.

### 3.3. Mechanical Test Results

#### 3.3.1. Bending Strength Results for Wool/Novolac Resin Panel Samples

Table 3 summarizes the results of the bending tests for the three types of composites tested.

A clear strengthening effect of the phenolic novolac resin matrix was observed, as its presence improves the mechanical performance of the wool–fiber composite by binding the fibers together and enabling effective stress transfer between them. According to the literature, the tensile strength of individual wool fibers is typically in the range of 120–174 MPa [25]. This means that the best-performing wool mat produced in this study, with a tensile strength of approximately 15 MPa, is about eight times weaker in mechanical terms than the fibers themselves. This reduction arises from the highly crimped and tortuous arrangement of fibers within the mat: under load, the applied force first straightens the fibers and only then leads to their shearing and failure. Nevertheless, a tensile strength on the order of 15 MPa is sufficient to ensure shape stability of the product and enables its use as a ceiling and wall insulation layer without the risk of detachment or sagging under gravity. The flexural modulus values in the range of approximately 0.84–1.43 GPa and flexural strength of 48–52 MPa obtained in this study for panels containing novolac resin (MW354, MWP) are significantly higher than the typical values reported for lightweight sheep wool-based insulation elements, in which the fibers mainly provide a continuous, highly porous core for thermal and acoustic purposes, rather than structural load-bearing capacity. For example, in a study on a sandwich panel with a sheep wool core designed primarily for acoustic performance, the recorded flexural strength was only 0.042 MPa at a maximum deflection of 7.5 mm, which illustrates the very limited load-bearing capacity of such systems [26]. On the other hand, the flexural strength values measured for our composites are lower than those reported for structural wool-reinforced epoxy composites. Semitekolos et al. [27] showed that the incorporation of up to 4.1 phr of wool fibers does not reduce the three-point flexural strength, which remains at about 136–141 MPa, similar to that of neat epoxy resin (around 137 MPa). For cementitious systems reinforced with sheep wool fibers, Ruiz-Díaz et al. [28] reported flexural strength values of approximately 5.5–8.2 MPa after 7 days of curing and, after 28 days, strengths comparable to mortars reinforced with synthetic fibers, which is similar to the level of about 4.5 MPa measured in the present work for the pure wool mat (MW), but clearly lower than the values achieved once the entangled fiber network is stiffened by the thermosetting novolac matrix. Taken together, these results indicate that the developed wool–novolac panels occupy an intermediate position between typical bio-based insulation composites and classical structural composites: they retain favorable thermal insulation and porous microstructure, while providing a flexural load-bearing capacity sufficient for self-supporting rigid insulation boards. Mechanical testing of neat novolac plates without wool fibers was not carried out in this study, because the resin was processed primarily as a binder for the fibrous mat rather than as a standalone structural material. For this reason, Table 3 focuses on the comparison between the pure wool mat and the corresponding wool/novolac composites.

#### 3.3.2. Shear Strength Results for Wool/Novolac Resin Panel Samples

Table 4 summarizes the shear test results for the tested panels made from sheep’s wool and the novolac matrix.

The test results clearly show a significant difference in the results of the dynamic tests. When the hammer strikes the composite, the novolac resin transmits the stress that appears suddenly and dissipates this type of stress most effectively. The presence of flame retardants impairs the dynamic properties, concentrating the stress on the ceramic filler throughout the entire volume of the sample. For the shear test table, the novolac matrix again shows a clear stiffening and energy-dissipation effect relative to the pure wool mat, and this trend is consistent with what is reported in the literature for wool-based composites under shear-like or dynamic loading. In the present study, the elastic modulus in shear Ef increases from only about 0.72 MPa for the pure wool mat (MW) to approximately 18 MPa for the flame-retardant composite (MW3_54) and 25 MPa for the composite with neat novolac (MWP), while the corresponding strain at break efB decreases from roughly 105% to 50% and 30%, respectively, and the work done at break WfB rises from about 246 N·mm to nearly 2850 N·mm and 4350 N·mm. Such a combination of higher stiffness, reduced ultimate strain, and strongly increased fracture work is characteristic of systems in which a continuous thermoset matrix constrains fiber sliding and transforms the originally very compliant, highly deformable fibrous network into a tougher, more energy-absorbing composite. Similar behavior has been observed in wool-reinforced cementitious and gypsum composites [29], where the incorporation of sheep wool fibers substantially increases flexural toughness and post-cracking energy absorption even when the nominal strength or modulus of the matrix does not increase proportionally. In polymer matrices, recent studies on sheep wool-filled epoxy and polyurethane and polyester resins have also emphasized the strong vibration-damping and energy-dissipation capacity of wool-based composites. The addition of a few percent of wool shifts resonance peaks and enhances mechanical damping compared with neat polymers, which is qualitatively in line with the high WfB values measured here for wool–novolac panels under impact-type shear loading. At the same time, Charpy impact tests on wool/epoxy composites show that, when the reference is a dense, stiff neat resin, adding wool fibers tends to reduce the absorbed impact energy to only about 7–25% of the matrix value depending on fiber length and content; in contrast, when the reference is a very soft fibrous mat, as in our case, impregnating the wool network with novolac increases the shear modulus by nearly two orders of magnitude and the work to failure by more than an order of magnitude, indicating that the phenolic matrix efficiently redistributes and dissipates dynamically applied shear stresses within the porous wool skeleton [30].

### 3.4. Thermal Analysis (TGA/DSC)

#### 3.4.1. Differential Scanning Calorimetry (DSC) Tests

**Figure 8:**
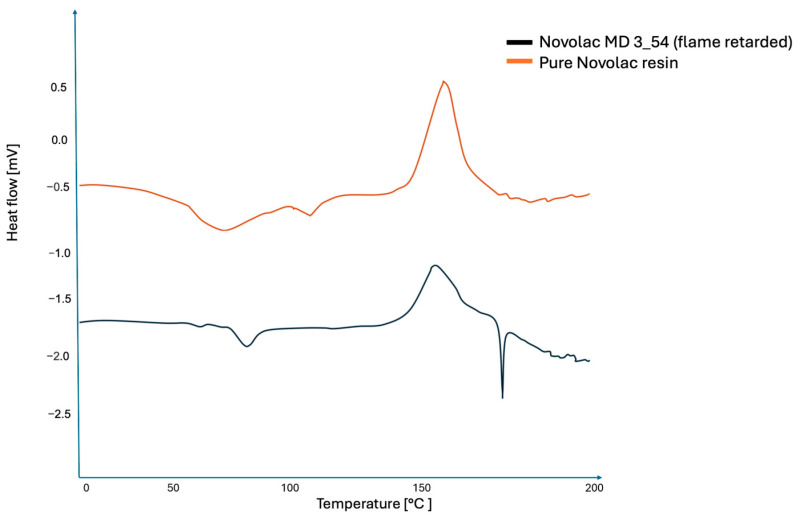
DSC curve for pure novolac resin and modified novolac MD 3_54 resin with flame retardant additive.

**Figure 9:**
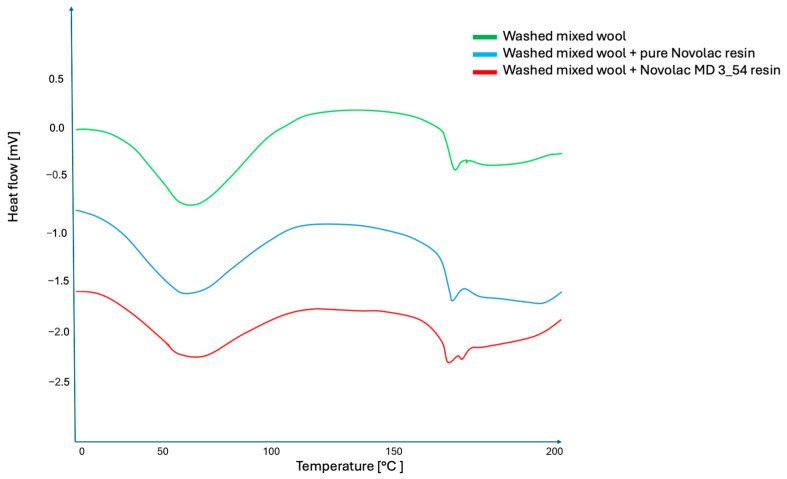
DSC curve for washed mixed wool and three types of composites.

Figure 8 and Figure 9 show the DSC curves for the resins and for the three types of composites.

DSC studies have shown the temperature ranges at which novolac resin crosslinks, releasing energy into the system. The crosslinking of novolac resins is associated with the release of gases (decomposition products), which is evident in the DSC curves. The differences between flame-retardant and non-flame-retardant resins are significant. Pure, non-flame-retardant resin contains more liquid phase. The crosslinking energies are similar in both cases. The DSC parameters obtained for the neat novolac resin in this study—an initial crosslinking temperature of about 140 °C and a curing enthalpy of approximately 48.6 J/g—are in good agreement with data reported for commercial phenol–formaldehyde novolacs. Domínguez et al. [31] compared the cure of novolac and resol phenolic resins and found that typical novolac systems exhibit a single dominant exothermic peak, with a maximum in the range of roughly 150–170 °C at heating rates of 5–10 °C/min, accompanied by reaction enthalpies in the order of a few tens of J/g, which is very close to the exotherm characteristics observed here. Kinetic DSC studies of powdered phenolic novolacs have similarly reported curing exotherms between about 130 and 180 °C, with activation energies in the range 70–100 kJ/mol and integral heats of reaction around 40–60 J/g, confirming that the curing window and calorimetric response of the novolac used in this work are representative of industrial phenolic systems.

DSC testing of the entire panels revealed that panels containing wool absorb moisture from the environment. This is a characteristic of wool that has a positive impact on its use in terms of insulation and user comfort. It maintains a consistent level of humidity in the air, preventing living spaces from becoming excessively dry. Wool begins to degrade above 200 °C, according to Figure 9. Above this temperature, rapid protein denaturation occurs, leading to the destruction of its structure.

The DSC studies revealed characteristic crosslinking temperature ranges for novolac resin and the hygroscopic behavior of wool in composites. For pure novolac resin, the onset temperature was 139.82 °C, with a crosslinking energy of 48.62 J/g, while for MD 354 resin it was 137.99 °C and 42.55 J/g, respectively, indicating similar crosslinking energies for both variants. In the DSC curves of the wool-containing samples, small endothermic effects were observed in the 70–80 °C range, which can be attributed to the evaporation of residual moisture in the hygroscopic keratin fibers. These features were minimized by prior oven-drying of the specimens and are therefore not used for quantitative assessment of water absorption, but they qualitatively confirm the tendency of wool to bind moisture. The initial degradation temperature of the wool in the composites was in the range of 215–218 °C (Figure 9), which confirms the thermal compatibility of the material with the novolac curing process carried out at 170—the fibers remain chemically and structurally intact during panel production.

The presence of flame-retardant additives in the MD 3_54 novolac slightly lowered the measured curing enthalpy (from 48.6 J/g to 42.6 J/g) without significantly shifting the onset temperature (137.99 °C compared to 139.82 °C for the pure novolac), which is consistent with the literature reports on filled or modified phenolic resins. In systems where novolac is blended with other thermosets [32]— or example, novolac/phthalonitrile or novolac/epoxy blends—the addition of a phenolic phase typically reduces the onset temperature of the main curing exotherm and may broaden or slightly decrease the peak height, but the total enthalpy per gram of reactive phenolic component remains in a similar range (≈30–40 J/g for 5–10 wt% novolac in phthalonitrile, compared to ≈25 J/g for neat phthalonitrile). The modest reduction in curing enthalpy observed for MD 3_54 can therefore be attributed to a lower effective fraction of crosslinkable phenolic units per unit mass and to partial replacement of organic resin by inorganic flame-retardant fillers, rather than to a fundamentally different curing mechanism.

Table 5 presents the numerical values from the DSC curves for the most significant phase transitions in all the samples tested.

The DSC results showed at what temperatures the novolac resin crosslinks and at what temperatures the crosslinking agents melt. Once the novolac matrix has fully crosslinked, it no longer exhibits a melting peak upon reheating; instead, only a glass transition is observed in subsequent scans, as is typical for phenolic thermosets.

#### 3.4.2. Thermogravimetric Analysis

**Figure 10:**
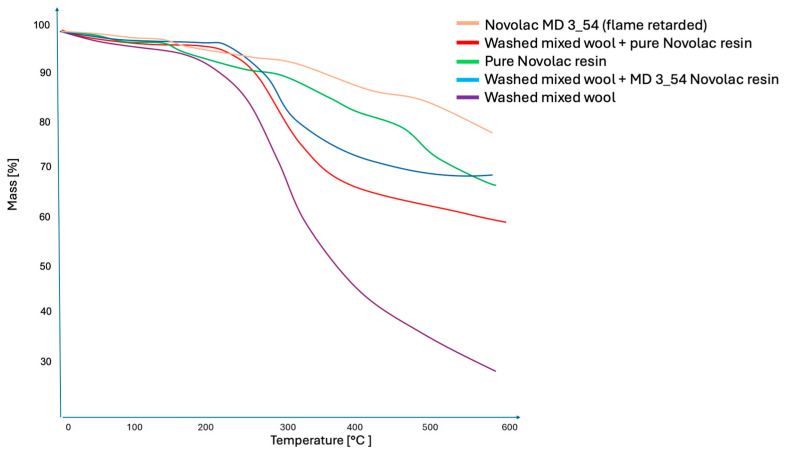
The thermogravimetric curve for pure wool, pure novolac resin, and the biocomposites.

Figure 10 shows the results of thermogravimetric analyses for pure wool, pure novolac resin, and the biocomposites tested in the project.

**Figure 11:**
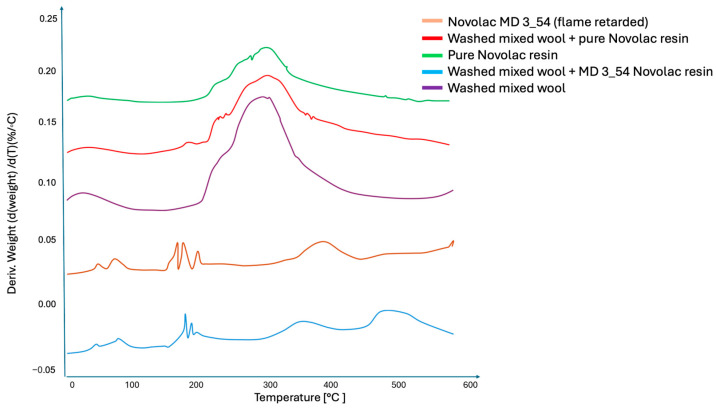
DTG curves for resin, wool, and composite samples.

Figure 11 shows the DTG curves for the resin, wool, and composite samples produced in this project.

Table 6 presents the results of thermogravimetric analysis of sheep’s wool, pure novolac resin, and composites based on wool and novolac resin.

The thermogravimetric studies revealed significant differences in thermal stability between pure sheep wool and its composites with novolac resin. The pure spun wool sample recorded a mass loss at 100 °C of 4.31% (related to moisture evaporation) and a loss at 170 °C of 5.68%, with a degradation onset temperature at 247 °C and a DTG peak temperature at 322 °C, with a residue of 27.68%. Impregnation with novolac resin significantly reduced the moisture content: in the MW composite (pure novolac resin), the loss at 100 °C was only 1.37%, and the residue after degradation increased to 62.14%. The best thermal stability was demonstrated by the composite with MD 354 resin containing a flame retardant. The mass loss at 100 °C was only 0.92%, the degradation onset temperature reached 251 °C, and the residue reached as much as 79.03%, which indicates effective thermal protection and increased pyrolysis resistance. For the wool–novolac composites, TGA revealed a slight reduction in moisture-related mass loss at 100 °C and a small shift in the degradation onset, together with a markedly higher char yield (above 60 wt% for the panel with pure novolac and nearly 80 wt% for the panel with flame-retardant novolac) compared with the raw wool. This behavior reflects the contribution of the phenolic matrix, which is known to form a thermally stable aromatic char and to increase the residue fraction in phenolic-based composites, especially when combined with inorganic flame retardants. Comparable trends have been observed in TGA studies of wool-reinforced thermoset biocomposites. The incorporation of wool does not significantly lower the onset temperature of the main degradation step, but the total mass loss profile is dominated by the decomposition of the matrix, and the final char content is higher than that of the neat polymer due to the charring propensity of both the phenolic network and the proteinaceous fiber phase [33]. Overall, the TGA results indicate that the developed wool/novolac panels possess thermal stability up to about 200 °C, in line with the literature data for wool and phenolic systems, and generate substantial char residues under inert conditions, which is favorable from the perspective of fire resistance and high-temperature performance. To disentangle the respective contributions of the phenolic matrix and the keratin fibers to the overall thermal stability and char yield, TGA was also performed on the neat novolac resin (Table 6). The resin exhibited an early onset of thermal degradation at around 180–200 °C, with the main DTG peak located close to 210 °C and a relatively low mass loss below 100 °C, reflecting only limited residual moisture and volatile content. Under nitrogen, the neat novolac left a substantial carbonaceous residue of approximately 68.5 wt% at 600 °C, which is typical of highly crosslinked phenolic systems and confirms their strong intrinsic charring tendency.

In comparison, the washed mixed wool started to decompose at somewhat higher temperatures (onset above 230 °C) but yielded a much lower char residue of about 28 wt%, consistent with the behavior of proteinaceous fibers that generate a nitrogen-rich but less abundant char. When wool was combined with the neat novolac resin, the composite panel showed intermediate characteristics: the onset of major degradation shifted towards higher temperatures relative to the neat resin, and the final residue was around 62 wt%, i.e., significantly higher than for wool alone but slightly lower than for the matrix by itself. For the composite containing the flame-retardant MD 3_54 novolac, both the onset of degradation and the maximum DTG peak were shifted to higher temperatures, and the char residue further increased to roughly 79 wt%. Taken together, these trends demonstrate that the high char yields observed for the wool/novolac panels arise from the combined charring of the phenolic matrix and the keratin fibers, while the flame-retardant formulation additionally stabilizes the residue and enhances the potential fire resistance of the composite.

### 3.5. Fiber Morphology (Laser/Optical Microscopy)

**Figure 12:**
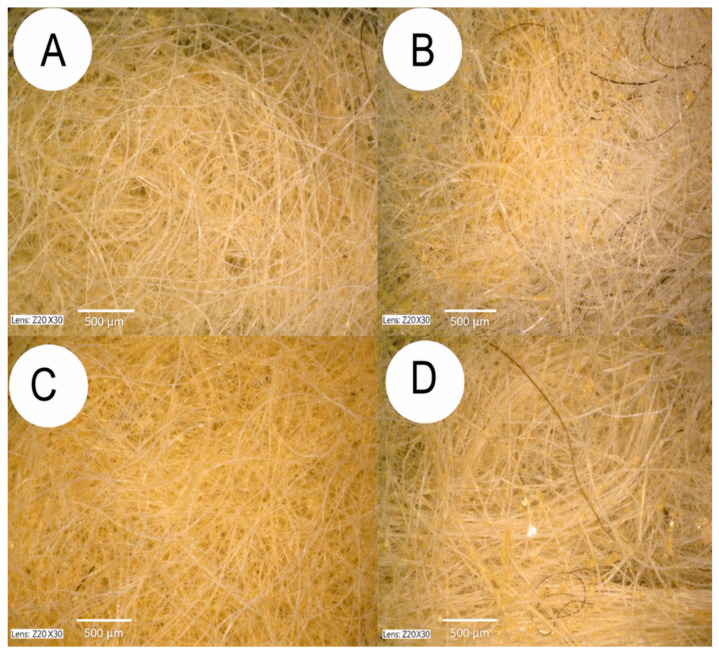
Photos of the composite material: washed sheep wool with the addition of pure novolac resin (**A**,**B**) and modified MD 3_54 (**C**,**D**) at approximately 30× magnification.

**Figure 13:**
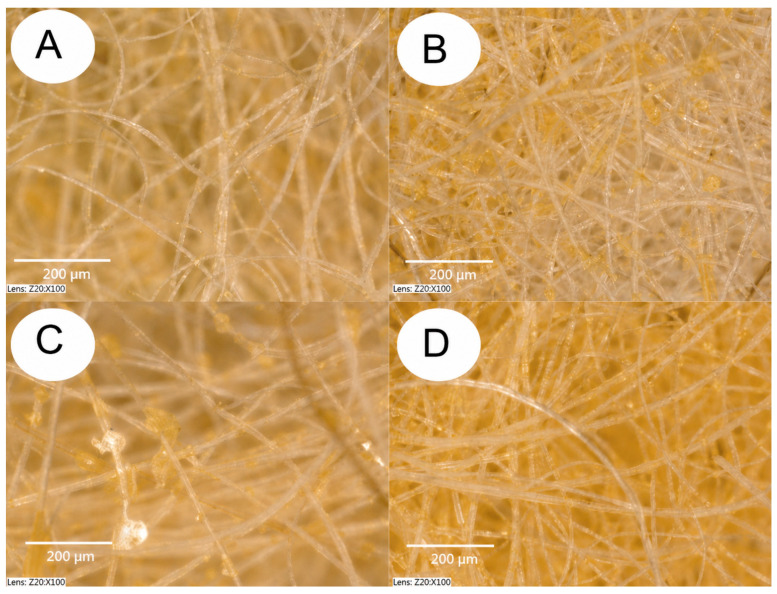
Photos of the composite material: washed sheep wool with the addition of pure novolac resin (**A**,**B**) and modified MD 3_54 (**C**,**D**) at approximately 100× magnification.

**Figure 14:**
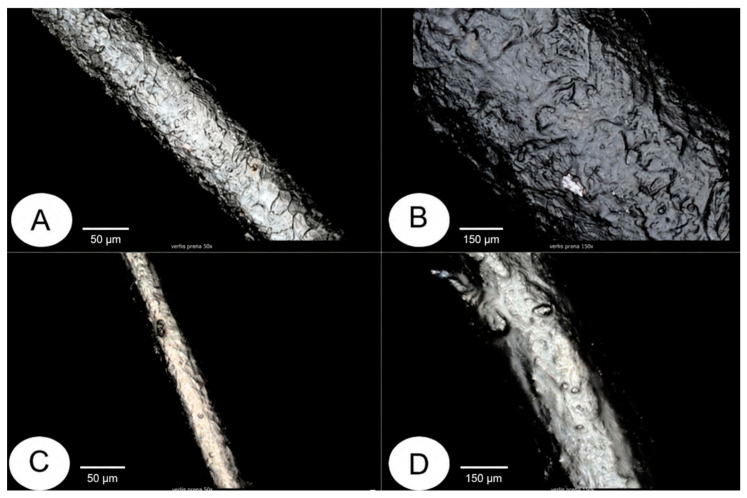
Photographs of a mixed sheep wool fiber washed under a laser microscope at approximately 50× magnification (**A**,**C**) and approximately 150× magnification (**B**,**D**).

**Figure 15:**
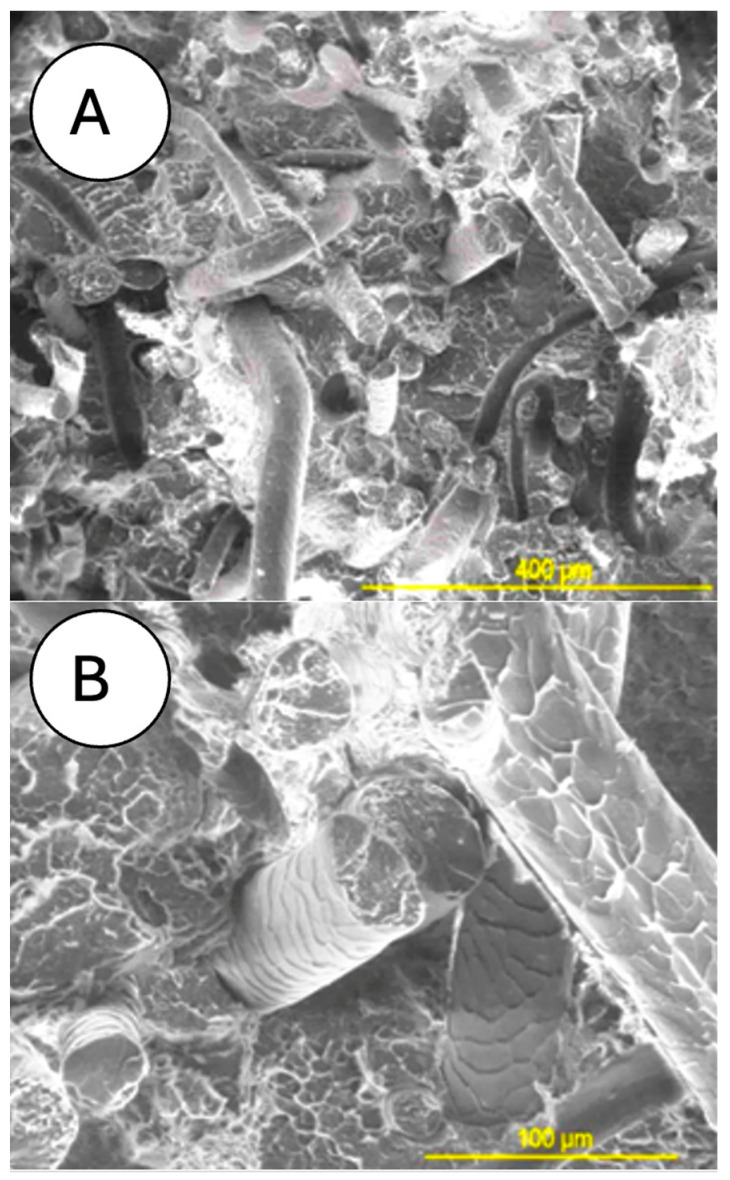
Image of composite under SEM microscope: wool fiber/pure novolac resin—magnifications of 350× (**A**) and 1000× (**B**).

To examine the morphology of the fibers in the prepared samples of the wool/novolac matrix composite, photographs were taken using a digital microscope at 30× (Figure 12) and 100× (Figure 13) magnification. To observe the structure of a single sheep wool fiber, photographs were taken using a laser microscope (Figure 14). In addition, photographs of the composite samples were taken using an SEM microscope for a more detailed analysis of the microstructures (Figure 15).

Optical micrographs (A–D) (Figure 12) of the sheep wool/novolac resin composite show a uniform distribution of fibers embedded in a continuous polymer matrix. The phenolic novolac resin forms a transparent phase that wets the wool fibers and creates thin interfacial layers around them, locally filling inter-fiber pores and producing resin bridges between neighboring filaments. Along the fiber–matrix interfaces, no pronounced gaps, deboned regions, or fiber pull-out traces are visible; instead, the resin closely follows the fiber contours, indicating good interfacial adhesion and effective mechanical interlocking between the wool fibers and the phenolic matrix. In panels B–D (Figure 13), resin-rich regions bind several fibers into compact bundles, which is expected to enhance stress transfer and restrict fiber slippage under load, while only isolated voids and impurities are observed, confirming satisfactory wetting of the fiber network.

Laser scanning micrographs (A–D) (Figure 14) show a single sheep wool fiber imaged using a reflected-light 3D laser microscope against a dark background. The fiber appears as an elongated cylindrical filament, and the characteristic overlapping cuticle scales are clearly visible along its length, forming an irregular, ridged surface pattern. Changes in magnification and illumination between images emphasize different aspects of the topography: from the overall fiber contour (C) to local surface relief and defects/damage on the scale edges (A, B, and D). This mode of laser microscopy provides high contrast between the bright fiber and the surrounding field, enabling precise determination of fiber diameter and quantitative assessment of surface roughness and scale morphology.

SEM micrographs of the sheep wool/novolac resin composite (Figure 15A,B) show a fracture surface after mechanical testing, characterized by a highly developed three-dimensional topography of the phenolic matrix with numerous protrusions and depressions. In both images, well-wetted, cylindrical wool fibers are embedded in a continuous matrix, with the resin closely surrounding a substantial portion of the fiber perimeter and locally forming collars and resin bridges, which indicates favorable interfacial adhesion and efficient stress transfer across the fiber–matrix interface. Short segments of pulled-out fibers and isolated micropores in the resin can be observed in some regions, potentially acting as crack initiators; however, the overall fracture morphology is consistent with relatively good fiber–matrix bonding.

### 3.6. Results of Thermal Conductivity Tests

Table 7 presents the results of the thermal conductivity measurements.

The measured thermal conductivity values confirm that the sheep wool/novolac panels operate in the typical range of bio-based fibrous insulations but remain less efficient than conventional expanded polystyrene foams. The measured thermal conductivity of the pure MW wool mat is 0.061 ± 0.005 W/(m·K), which is significantly higher than the typical range of 0.035–0.045 W/(m·K) reported for optimized sheep’s wool mats and felts, a difference that can be attributed to the higher bulk density and less optimized porous structure of the tested material. In contrast, impregnation with the phenolic novolac resin increases the thermal conductivity to 0.071 ± 0.005 W/(m·K) for MWP and 0.074 ± 0.006 W/(m·K) for MW3_54, reflecting the higher solid fraction, partial filling of pores, and formation of continuous resin bridges that facilitate conductive heat transfer through the solid phase. In terms of thermal insulation, the measured λ values for the MW wool mat (0.061 ± 0.005 W/(m·K)) and the MWP and MW354 composites (0.071 ± 0.005 and 0.074 ± 0.006 W/(m·K), respectively) are higher than the values typical for commercial fibrous materials, such as mineral wool or optimized sheep’s wool products, for which the literature reports a range of approximately 0.035–0.045 W/(m·K). Compared to expanded polystyrene (EPS), which at an average temperature of 10 °C exhibits a λ of 0.030–0.040 W/(m·K), the tested panels, therefore, require greater thickness to achieve the same thermal resistance. It should be emphasized, however, that wool/novolac panels combine the function of a load-bearing board with that of thermal insulation, whereas typical sheep’s wool and EPS mats serve exclusively as insulation materials [35]. When comparing these results with the literature data for commercial fibrous insulation materials (mineral wool, wood wool, and sheep’s wool), which typically have a λ value in the range of approximately 0.035–0.045 W/(m·K), it can be concluded that the tested wool/novolac panels exhibit moderately elevated thermal conductivity, but still fall within the broader range of materials used in building partitions with low requirements for minimum insulation layer thickness [35,36].

From a purely thermal point of view, the wool–novolac panels developed in this study exhibit higher thermal conductivity values (λ = 0.061 W/(m·K) for the pure wool mat and λ ≈ 0.071–0.074 W/(m·K) for the composites with novolac resin) than reference expanded polystyrene (EPS) foam (λ ≈ 0.040 W/(m·K)) [34]. Consequently, for a given thermal resistance, the required thickness of wool-based insulation is roughly 1.5–1.8 times larger than that of EPS, implying a higher material mass per square meter of wall area. On this basis alone, EPS appears more favorable in terms of operational energy savings and envelope compactness. The wool–novolac panels have a substantially higher solid content and apparent density than expanded polystyrene foams, where up to 98% of the volume is air. This difference in bulk density and solid fraction is an important factor behind the higher thermal conductivity of the composites compared with EPS, in addition to the intrinsically higher thermal conductivity of the phenolic matrix relative to the gas-filled closed cells of EPS. Compared with typical commercial sheep wool insulation products (λ ≈ 0.035–0.045 W·m^−1^·K^−1^), the higher thermal conductivity of the panels developed here can be explained mainly by their increased density and the presence of a continuous phenolic matrix. Impregnation with novolac resin reduces the total porosity and partially fills the air-filled inter-fiber voids, while forming rigid bridges between fibers that enhance solid-phase conduction. At the same time, the relatively high panel density required for sufficient mechanical performance limits the share of stagnant air in the microstructure, which is the most efficient insulating phase in fibrous materials. As a result, the wool/novolac boards occupy an intermediate position between lightweight wool felts and dense structural composites. They retain a largely porous architecture, but their solid skeleton is more developed than in conventional wool batts, leading to somewhat higher λ values for a given thickness. In principle, the thermal conductivity of wool-based rigid boards could be further reduced by employing alternative, lower-density matrices, such as polyurethane or foamed phenolic systems, which provide a larger volume fraction of closed gas cells. However, such matrices typically involve higher flammability or more complex blowing-agent chemistries, and may compromise the favorable char-forming behavior of the phenolic matrix and the intrinsically low flammability of wool reported in the literature, suggesting a potentially beneficial fire response in the wool/novolac panels, which, however, needs to be verified by dedicated reaction-to-fire and fire-resistance tests beyond the scope of this study.

Due to their brittleness and the difficulty of preparing sufficiently thick, homogeneous samples, no thermal conductivity measurements were performed for panels made of resin alone. Comparisons of λ values were limited to wool mats and wool/novolac composites, as well as to the literature data for phenolic foams and other fibrous insulation materials.

Published life-cycle assessment (LCA) studies on building insulation consistently show that bio-based materials can offer substantially lower embodied energy and global warming potential than conventional petrochemical foams when compared at the same functional unit, i.e., 1 m^2^ of wall providing a given thermal resistance. Comparative cradle-to-grave LCAs for wood fiber, hemp, flax and miscanthus insulation versus expanded polystyrene (EPS) and stone wool report that the best-performing bio-based products achieve markedly lower climate impacts, even though their thermal conductivity is often less favorable and larger thicknesses are required. At the same time, recent work on waste-derived thermal insulation materials indicates that using secondary raw materials can further reduce cradle-to-grave environmental burdens compared with both virgin mineral wool and EPS, provided that end-of-life scenarios are carefully managed. These findings are directly relevant to the present panels, where roughly half of the composite mass is low-grade sheep wool that would otherwise be treated as agricultural waste. Although the phenolic novolac binder is of petrochemical origin, the additional thickness required to match the thermal performance of EPS mainly increases the share of biogenic, waste-based fiber, so that the overall embodied energy and global warming potential per functional unit are expected to remain competitive or favorable with respect to EPS and mineral wool. A rigorous quantification of this expectation would, however, require a dedicated LCA of the specific wool–novolac system, which lies beyond the scope of this study [36,37,38,39].

End-of-life scenarios further accentuate these differences. For EPS, current practice is dominated by landfilling or incineration with limited material recycling, and the climate impact of incineration is governed entirely by fossil carbon. Wool–novolac panels cannot be regarded as fully biodegradable due to the thermoset matrix, but roughly 50 wt% of their mass is a biogenic component, so incineration with energy recovery partially substitutes fossil fuels and shifts a significant share of emissions into the biogenic carbon pool. Comparative LCAs of natural-fiber insulating boards made from waste or agricultural by-products show that such products often achieve the lowest cradle-to-grave energy demand and global warming potential among examined boards, even when their thermal conductivity is less favorable than that of EPS. Overall, this suggests that, despite requiring increased thickness to meet a given U-value, sheep wool/novolac panels are competitive with EPS in terms of life-cycle energy demand. While no experimental data on biodegradability, recyclability, or cradle-to-grave impacts were generated in this work, the partial substitution of fossil-based polymer by a biogenic fiber phase and the valorization of low-value wool streams align with current circular-economy strategies in the building sector. Future studies should quantify these potential benefits through dedicated life-cycle assessment and end-of-life scenario analysis.

## 4. Conclusions

The physicochemical characterization of mixed Polish sheep wool confirmed a keratinous protein structure rich in carbon, nitrogen, and sulphur, with only trace amounts of toxic elements, indicating that this low-value fiber stream is suitable as a bio-based reinforcement for insulation panels from both a performance and environmental standpoint. FTIR, laser microscopy, and SEM showed that phenolic novolac resin wets and encapsulates the wool fibers effectively, forming continuous collars and bridges and providing good fiber–matrix adhesion that underpins efficient stress transfer in the composite panels.

Mechanical testing demonstrated that impregnation with novolac transforms the compliant wool mat into a rigid, yet still lightweight, panel. The flexural modulus and strength increased by almost an order of magnitude, while shear modulus and work to failure under impact-type loading rose by more than an order of magnitude compared with the pure wool mat, positioning the best formulations between typical bio-based insulation boards and structural fiber composites. The thermal conductivity of the developed materials (λ = 0.061 W·m^−1^·K^−1^ for the wool mat and λ ≈ 0.071–0.074 W·m^−1^·K^−1^ for the wool–novolac panels) is higher than that of expanded polystyrene (≈0.040 W·m^−1^·K^−1^), but remains within the range reported for commercial sheep wool and other natural-fiber insulation products, confirming their suitability as thermal insulants for walls and ceilings where somewhat larger thicknesses are acceptable.

DSC and TGA analyses confirmed that the novolac resin cures in a temperature and enthalpy range typical of industrial phenolic systems and that wool fibers remain thermally stable throughout the curing schedule, beginning to degrade only above about 200–220 °C. The high char yields observed for the wool–novolac panels under nitrogen reflect the combined charring of the phenolic matrix and the keratin fibers and are generally considered beneficial for high-temperature stability; however, they cannot be directly translated into standardized fire-resistance ratings without additional testing.

From a life-cycle perspective, combining an annually renewable, low-value wool by-product with a phenolic binder can potentially reduce the embodied energy and global warming potential of insulation boards compared with purely petrochemical foams, as suggested by published LCAs on natural-fiber insulation materials. A quantitative assessment of these aspects for the present panels was beyond the scope of this study and should be addressed in future work.

Overall, the results show that sheep wool/novolac panels provide a balanced combination of microstructural integrity, mechanical robustness, thermal insulation capability and favorable thermochemical behavior, making them promising candidates for sustainable rigid insulation boards in energy-efficient building envelopes. Future work should address long-term hygrothermal ageing, fire testing under standardized furnace conditions, and optimization of panel density and binder content to further improve the trade-off between thermal performance, mechanical properties, and environmental impact.

## Figures and Tables

**Table 1:** Element content in washed sheep wool fibers.

Element	Element Content in Washed Sheep Wool Fibers [mg/kg]
Aluminum (Al)	27.23
Arsenic (As)	0.14
Calcium (Ca)	2003.42
Iron (Fe)	65.02
Potassium (K)	1016.68
Magnesium (Mg)	339.96
Phosphorus (P)	113.07
Lead (Pb)	0.43
Sulphur (S)	18,315.96
Zinc (Zn)	72.75

**Table 2:** Nitrogen and carbon content in washed sheep wool fibers.

Element	Element Content in Washed Sheep Wool Fibers [%]
Nitrogen (N)	14.82
Carbon (C)	47.80

**Table 3:** Bending strength results for wool/novolac resin panel samples.

Sample	E_f_	S_x1_	S_x2_	S_x3_	S_fM_	e_fM_
	[MPa]	[MPa]	[MPa]	[MPa]	[MPa]	[%]
Washed mixed wool + pure novolac resin (MWP)	1425.50 +/− 71.28	15.66 +/− 0.78	30.63 +/− 1.53	45.03 +/− 2.25	51.75 +/− 2.59	4.30 +/− 0.22
Washed mixed wool (MW)	117.29 +/− 5.86	1.40 +/− 0,07	2.77 +/− 0.14	4.05 +/− 0.20	4.45 +/− 0.22	3.36 +/− 0.17
Washed mixed wool + MD 3_54 Novolac resin (MW3_54)	843.73 +/− 42.19	10.09 +/− 0.50	23.85 +/− 1.19	37.72 +/− 1.89	48.16 +/− 2.41	4.37 +/− 0.22

E_f_—flexural modulus; S_x1_, S_x2_, and S_x3_—flexural stress at three selected mid-span deflections; S_fM_—flexural strength at maximum load; e_fM_—flexural strain at maximum load.

**Table 4:** Shear strength results for wool/novolac resin panel samples.

Sample	E_f_	E_fb_	W_fB_
	[MPa]	[%]	[Nmm]
Washed mixed wool + MD 3_54 novolac resin (MW3_54)	18.00 +/− 0.90	50.15 +/− 2.51	2854.97 +/− 142.75
Washed mixed wool + pure novolac resin (MWP)	25,00 +/− 1.25	30.22 +/− 1.51	4346.06 +/− 217.303
Washed mixed wool (MW)	0.72 +/− 0.04	105.23 +/− 5.26	245.88 +/− 12.29

E_f_ is the elastic modulus, E_fB_ is the elongation at break, and W_fB_ is the work done at break.

**Table 5:** Analysis of DSC curves of manufactured materials.

Sample Name	Temp. Max Peak of Dehydration Wool [°C]	Heat of Evaporation [J/g]	Temp. of Start Degradation [°C]	Resin Initial Crosslinking Temperature [°C]	Resin Crosslinking Energy [J/g]	Melting Start Temperature [°C]	Max Melting Energy of Resin [J/g]
Pure Novolac resin	-	-	-	139.82	48.62	45.33	18.31
Novolac MD 3_54	-	-	-	137.99	42.55	50.06	8.80
Washed mixed wool	78.56	173.74	218.58	-	-	-	-
Washed mixed wool + pure Novolac resin	72.77	175.34	215.17	-	-	-	-
Washed mixed wool + MD 3_54 Novolac resin	76.04	191.20	216.19	-	-	-	-

**Table 6:** The thermogravimetric analysis of the samples produced.

Sample Name	Mass Loss at Temp. 100 °C [%]	Weight Loss During Processing [%]	Degradation Onset Temperature [°C]	Temperature of the Onset of Major Degradation [°C]	Maximum DTG Peak of the Degradation Process [°C]	Residue [%]
Washed mixed wool	4.31	5.68	247.15	229.11	322.55	27.68
Pure novolac resin	0.74	2.22	202.11	180.85	208.09	68.50
Novolac MD 3_54	0.78	2.20	204.32	189.34	210.53	70.21
Washed mixed wool + pure novolac resin	1.37	2.27	241.44	188.59	331.19	62.14
Washed mixed wool + MD 3_54 novolac resin	0.92	1.42	251.13	236.91	329.42	79.03

**Table 7:** Thermal conductivity results for wool/novolac resin panel samples.

Sample	Thermal Conductivity
	[W/m*K]
Washed mixed wool + pure novolac resin	0.071 +/− 0.005
Washed mixed wool	0.061 +/− 0.005
Washed mixed wool + MD 3_54 novolac resin	0.074 +/− 0.006
Expanded polystyrene	0.04 [34]

## Data Availability

The original contributions presented in this study are included in the article. Further inquiries can be directed to the corresponding authors.

## References

[1] Caggiano A. (2023). Energy in Construction and Building Materials. Materials.

[2] Vėjelis S., Vaitkus S., Skulskis V., Kremensas A., Kairytė A. (2024). Performance Evaluation of Thermal Insulation Materials from Sheep’s Wool and Hemp Fibres. Materials.

[3] Garcia J.F., Kranzl L. (2018). Ambition Levels of Nearly Zero Energy Buildings (nZEB) Definitions: An Approach for Cross-Country Comparison. Buildings.

[4] Zandonella Callegher C., Grazieschi G., Wilczynski E., Oberegger U.F., Pezzutto S. (2023). Assessment of Building Materials in the European Residential Building Stock: An Analysis at EU27 Level. Sustainability.

[5] Superti V., Forman T.V., Houmani C. (2021). Recycling Thermal Insulation Materials: A Case Study on More Circular Management of Expanded Polystyrene and Stonewool in Switzerland and Research Agenda. Resources.

[6] Neri M., Pilotelli M., Traversi M., Levi E., Piana E.A., Bannó M., Cuerva E., Pujadas P., Guardo A. (2021). Conversion of End-of-Life Household Materials into Building Insulating Low-Cost Solutions for the Development of Vulnerable Contexts: Review and Outlook towards a Circular and Sustainable Economy. Sustainability.

[7] Olsø B.G., Haukø A.M., Risholt B. (2024). Experimental Study of Fire Exposed Expanded Polystyrene (EPS) Insulation Protected by Selected Coverings. Heliyon.

[8] Dénes T.-O., Iştoan R., Tǎmaş-Gavrea D.R., Manea D.L., Hegyi A., Popa F., Vasile O. (2022). Analysis of Sheep Wool-Based Composites for Building Insulation. Polymers.

[9] Hegyi A., Bulacu C., Szilagyi H., Lăzărescu A.V., Meiţă V., Vizureanu P., Sandu M. (2021). Improving Indoor Air Quality by Using Sheep Wool Thermal Insulation. Materials.

[10] Korjenic A., Klarić S., Hadžić A., Korjenic S. (2015). Sheep Wool as a Construction Material for Energy Efficiency Improvement. Energies.

[11] Szczecina J., Szczepanik E., Barwinek J., Szatkowski P., Niemiec M., Zhakypbekovich A.I., Molik E. (2025). Sheep Wool as Biomass: Identifying the Material and Its Reclassification from Waste to Resource. Energies.

[12] Hegyi A., Vermeșan H., Lăzărescu A.-V., Petcu C., Bulacu C. (2022). Thermal Insulation Mattresses Based on Textile Waste and Recycled Plastic Waste Fibres, Integrating Natural Fibres of Vegetable or Animal Origin. Materials.

[13] Fischer H., Korjenic A. (2023). Hygrothermal Performance of Bio-Based Exterior Wall Constructions and Their Resilience under Air Leakage and Moisture Load. Buildings.

[14] Vėjelis S., Skulskis V., Kremensas A., Vaitkus S., Kairytė A. (2023). Performance of Thermal Insulation Material Produced from Lithuanian Sheep Wool. J. Nat. Fibers.

[15] Kicińska-Jakubowska A., Broda J., Zimniewska M., Bączek M., Mańkowski J. (2023). Effect of Blend Composition on Barrier Properties of Insulating Mats Produced from Local Wool and Waste Bast Fibres. Materials.

[16] Parlato M.C.M., Porto S.M.C. (2020). Organized Framework of Main Possible Applications of Sheep Wool Fibers in Building Components. Sustainability.

[17] Galaska M.L., Sqrow L.D., Wolf J.D., Morgan A.B. (2019). Flammability Characteristics of Animal Fibers: Single Breed Wools, Alpaca/Wool, and Llama/Wool Blends. Fibers.

[18] Mattiello S., Guzzini A., Del Giudice A., Santulli C., Antonini M., Lupidi G., Gunnella R. (2023). Physico-Chemical Characterization of Keratin from Wool and Chicken Feathers Extracted Using Refined Chemical Methods. Polymers.

[19] Richter M., Horn W., Juritsch E., Klinge A., Radeljic L., Jann O. (2021). Natural Building Materials for Interior Fitting and Refurbishment—What about Indoor Emissions?. Materials.

[20] Cheng X.-W., Guan J.-P., Chen G., Yang X.-H., Tang R.-C. (2016). Adsorption and Flame Retardant Properties of Bio-Based Phytic Acid on Wool Fabric. Polymers.

[21] Atbir A., Taibi M., Aouan B., Khabbazi A., Ansari O., Cherkaoui M., Cherradi T. (2023). Physicochemical and Thermomechanical Performances Study for Timahdite Sheep Wool Fibers Application in the Building’s Insulation. Sci. Rep..

[22] Sun M., Chen M., Li S., Dai C., Chen Y. (2022). Study on Structure and Properties of Hu Sheep Wool. J. Nat. Fibers.

[23] Gül S., Dikme M. (2023). Investigation of Some Physical and Morphological Characteristics of Wool of Malya Sheep. J. Nat. Fibers.

[24] Adnan M.A. (2019). Synthesis of Polyurethane Based on (Resol Novolac Resin-Polyethylene Glycol) Copolymer and Their Analytical Study. J. Kufa-Phys..

[25] Szatkowski P., Barwinek J., Zhakypbekovich A.I., Szczecina J., Niemiec M., Pielichowska K., Molik E. (2025). The Use of Sheep Wool Collected from Sheep Bred in the Kyrgyz Republic as a Component of Biodegradable Composite Material. Appl. Sci..

[26] Tiuc A. (2020). A Novel Acoustic Sandwich Panel Based on Sheep Wool. Coatings.

[27] Semitekolos D., Pardou K., Georgiou P., Koutsouli P., Bizelis I., Zoumpoulakis L. (2021). Investigation of Mechanical and Thermal Insulating Properties of Wool Fibres in Epoxy Composites. Polym. Polym. Compos..

[28] Jóźwiak-Niedźwiedzka D., Fantilli A.P. (2020). Wool-Reinforced Cement Based Composites. Materials.

[29] Pederneiras C.M., Veiga R., de Brito J. (2019). Rendering Mortars Reinforced with Natural Sheep’s Wool Fibers. Materials.

[30] Tripathy P., Biswas S. (2022). Mechanical and Thermal Properties of Mineral Fiber Based Polymeric Nanocomposites: A Review. Polym.-Plast. Technol. Mater..

[31] Domínguez J.C., Alonso M.V., Oliet M., Rojo E., Rodríguez F. (2010). Kinetic Study of a Phenolic-Novolac Resin Curing Process by Rheological and DSC Analysis. Thermochim. Acta.

[32] Zhang H., Wang B., Wang Y., Zhou H. (2020). Novolac/Phenol-Containing Phthalonitrile Blends: Curing Characteristics and Composite Mechanical Properties. Polymers.

[33] Sadroddini M. (2026). Enhanced Mechanical and Thermal Properties of Polyester/Glass/Sheep Wool Fiber Hybrid Composites by Successful Replacement Glass with Wool. Sci. Rep..

[34] Simpson A., Rattigan I., Kalavsky E., Parr G. (2020). Thermal Conductivity and Conditioning of Grey Expanded Polystyrene Foams. Cell. Polym..

[35] Awoyera P.O., Olofinnade O., Olojede S., Onyelowe K. (2025). Enhancing the Thermal Performance of Expanded Polystyrene with Radiant Barrier Foil Boards for Improved Building Insulation. Sci. Rep..

[36] Hill C., Norton A., Dibdiakova J. (2018). A Comparison of the Environmental Impacts of Different Categories of Insulation Materials. Energy Build..

[37] Füchsl S., Rheude F., Röder H. (2022). Life Cycle Assessment (LCA) of Thermal Insulation Materials: A Critical Review. Clean. Mater..

[38] Schulte M., Lewandowski I., Pude R., Wagner M. (2021). Comparative life cycle assessment of bio-based insulation materials: Environmental and economic performances. GCB Bioenergy.

[39] Islam S., Bhat G., Mani S. (2024). Life cycle assessment of thermal insulation materials produced from waste textiles. J. Mater. Cycles Waste Manag..

